# Supplementary material for: The interactome of intact mitochondria by cross-linking mass spectrometry provides evidence for coexisting respiratory supercomplexes
Source: Mol Cell Proteomics. 2017 Dec 8;17(2):216–32. doi: 10.1074/mcp.RA117.000470 (PMC5795388; doi:10.1074/mcp.RA117.000470)
Supplement: Supplemental Data [file supp_RA117.000470_133922_0_supp_23978_fzffwf.zip › spectra_annotation/mito_spectra_annotation/223-1-3-1-3-1.pdf]

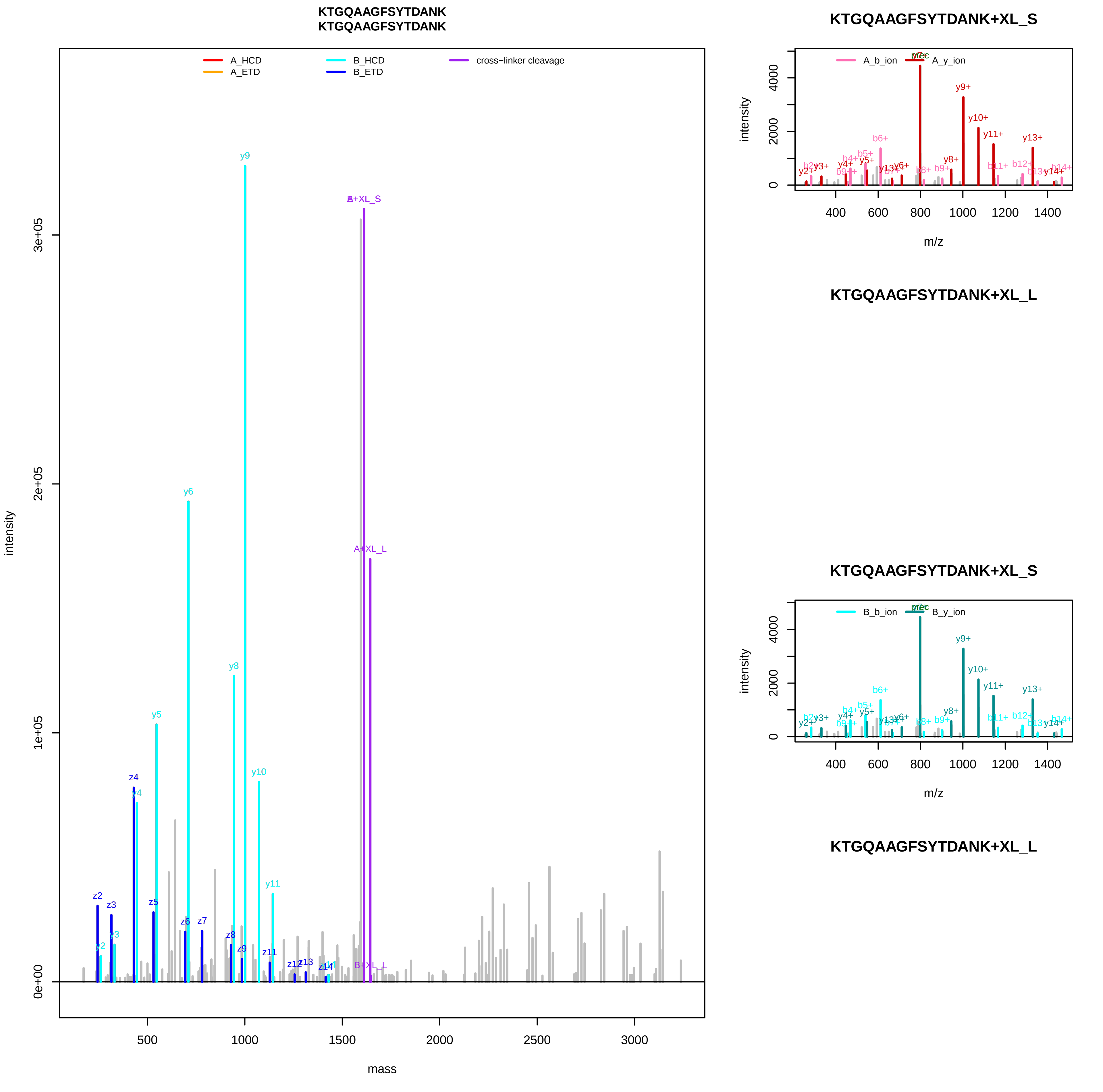
KTGQAAGFSYTDANK
KTGQAAGFSYTDANK
A_HCD
A_ETD
B_HCD
B_ETD
cross−linker cleavage
intensity
mass
0e+00
1e+05
2e+05
3e+05
500
1000
1500
2000
2500
3000
A+XL_S
A+XL_L
B+XL_L
y2
y3
y4
y5
y6
y8
y9
y10
y11
z2
z3
z4
z5
z6
z7
z8
z9
z11
z12
z13
z14
KTGQAAGFSYTDANK+XL_S
A_b_ion
A_y_ion
intensity
m/z
0
2000
4000
400
600
800
1000
1200
1400
prec
y9+
y10+
y11+
y13+
b6+
b5+
y5+
y4+
y3+
b2+
y2+
y8+
b9+
b11+
b12+
y14+
b14+
KTGQAAGFSYTDANK+XL_L
KTGQAAGFSYTDANK+XL_S
B_b_ion
B_y_ion
intensity
m/z
0
2000
4000
400
600
800
1000
1200
1400
prec
y9+
y10+
y11+
y13+
b6+
b5+
y5+
y4+
y3+
b2+
y2+
y8+
b9+
b11+
b12+
y14+
b14+
KTGQAAGFSYTDANK+XL_L
